# Correlative Transcriptome and Metabolome Analysis of the Maize Shoot Response to Salt Stress

**DOI:** 10.3390/plants14233554

**Published:** 2025-11-21

**Authors:** Wangdan Xiong, Lingxin Zhang, Yujian Wang, Guo Wei, Kaikai Zhu, Kai Zhao, Zhenying Wu

**Affiliations:** 1 College of Life Sciences, Anqing Normal University, Anqing 246011, China; 2 College of Agriculture, Qingdao Agricultural University, Qingdao 266109, China; 3 College of Horticulture and Landscape Architecture, Yangzhou University, Yangzhou 225009, China; 4 Co-Innovation Center for Sustainable Forestry in Southern China, Nanjing Forestry University, Nanjing 210037, China

**Keywords:** salt stress, multi-omics integration, regulatory networks, secondary metabolism, *Zea mays*

## Abstract

Soil salinity increasingly jeopardizes maize productivity. Although previous studies have documented maize physiological responses under salt stress, the integrated regulatory networks linking signal perception, transcriptional reprogramming, and metabolic adjustment in shoots remain poorly understood. Here, we combined phenotypic, physiological, enzymatic, transcriptomic, and metabolomic analyses to systematically dissect maize seedling leaf responses to NaCl. Salt stress significantly inhibited photosynthesis, reduced plant biomass, and disturbed ion homeostasis, as evidenced by increased Na^+^/K^+^ ratio, elevated MDA level, and enhanced antioxidant enzyme activities (SOD, CAT, POD). Through transcriptomic profiling analysis, 1558 DEGs were identified, which were predominantly associated with MAPK and hormone signal transduction and secondary metabolism. Among the DEGs, transcription factors (AP2, bHLH, bZIP, MYB, NAC, WRKY) showed marked expression changes. Moreover, metabolomic analysis detected 232 DAMs, spanning amino acids and derivatives, phenolic acids, alkaloids, organic acids, and lipids. Integrated omics revealed that salt stress induced widespread transcriptional reprogramming of signaling genes, which was correlated with metabolic adjustments favoring osmolyte accumulation, antioxidant biosynthesis, and membrane stabilization. These findings provide a comprehensive multi-omics resource for understanding maize shoot responses to salinity and highlight potential targets to breed salt-tolerant varieties.

## 1. Introduction

Soil salinization, a widespread environmental challenge in agriculture, reduces crop yield and limits silage production [1]. Salt accumulation in soil creates an osmotic gradient that restricts water uptake by plant roots, leading to osmotic stress. Under salt stress, plants take up Na^+^ through the root system and then transport it to shoots [2]. Excessive accumulation in plants exerts cytotoxic effects [2]. Elevated Na^+^ concentrations in shoots disrupt photosynthetic carbon assimilation and ultimately reduce crop yield [3]. Salt stress also promotes reactive oxygen species (ROS) overaccumulation, inflicting oxidative harm on essential cellular components (such as proteins, lipids, and nucleic acids, etc.) [4]. These combined effects disturb cellular homeostasis and interfere with essential metabolic pathways, thereby threatening plant survival and agricultural yield [2]. Therefore, the strategic use of saline lands and the improvement of crop salt tolerance are critical to global food security and promoting ecological restoration [5].

Maize (*Zea mays* L.), a fundamental cereal and vital silage crop, is widely cultivated across diverse agroecological zones [6,7]. Soil salinity restricts maize growth throughout its developmental stages and results in substantial yield losses [8]. The crop exhibits vulnerability to salt stress during early growth [6,7]. Salt stress reduces reduced germination rates and restricts seedling development, and yield losses may exceed 30% in severely affected areas [6,7]. Therefore, breeding salt-tolerant maize varieties is essential for sustaining global production under increasing soil salinization.

Plants exposed to saline conditions develop multiple adaptive mechanisms that mitigate toxicity and support survival [9,10,11]. Earlier studies examined these adaptive features through morphological, physiological, and biochemical perspectives. Physiological research shows that maize responds to salt stress through osmotic adjustment (e.g., proline accumulation) and antioxidant enzyme activation [6,12,13]. Transcriptomic and metabolomic analyses show dynamic responses triggered by salt stress [7,14,15,16]. In rice, transcriptomic research indicated that antioxidative and osmotic pathways, sucrose and starch metabolism, glutathione metabolism, and flavonoid biosynthesis are central to salt stress tolerance [17]. Studies in major crops have also identified key regulators, including SOS1 ion transporters and ABA-dependent transcriptional regulators such as NAC and MYB [12,18]. Metabolomic studies show extensive reprogramming of compatible solutes (e.g., sugars, polyamines) and secondary metabolites (e.g., flavonoids, alkaloids) under salt stress [19]. Multi-omics technologies have recently advanced the systematic study of stress-responsive networks. Emerging evidence shows that plants adapt to saline environments through complex regulatory processes that span early signal perception and downstream metabolic adjustments. Mitogen-activated protein kinase (MAPK) cascades and hormone signal transduction function as central hubs in sensing and relaying salt signals [20]. MAPK cascades have conserved signaling roles in eukaryotes, which contain three types of protein kinases (MAPK, MAPKK, and MAPKKK) [21,22]. Research reports that MAPK cascades respond to salt stress in maize roots and that *ZmMKK1* and *ZmMKK4* play positive roles in maize salt tolerance [23,24,25]. At the metabolic level, salt stress induces significant metabolic alterations, many of which function as osmolytes, antioxidants, or signaling molecules to maintain cellular homeostasis [26]. A recent multi-omics analysis identified the regulation of flavonoid biosynthesis in roots as important to the adaptation of maize salt–alkali stress adaptation [27]. Roots are the initial tissue to sense salt, whereas shoots also contribute critically to systemic acclimation and overall tolerance [2,9,28]. The molecular mechanisms that govern these responses in maize shoots remain fragmented. Although one study found that salt-tolerant maize accumulated starch, sucrose, linoleic acid, and phenylpropanoid derivatives as key medium-term (8-day) responses, the longer-term coordination between transcriptional regulation and metabolic adaptation remains unclear [29]. Here, we performed 12-day salt treatment of maize shoots and used integrated multi-omics profiling to systematically dissect the coordinated regulatory networks for salt tolerance. This timeframe was selected based on recent maize research showing that 12-day treatment yields the broadest phenotypic variation for assessing saline–alkali tolerance [27] and distinguishes tolerant and sensitive hybrids through antioxidant and ionic responses [7]. The extended duration also captures stable acclimation processes and cumulative cellular damage, which aligns with physiological and omics-based measurements.

This study integrates transcriptomic and metabolomic data to characterize maize shoot responses to salt stress. The objectives were to identify differentially expressed genes (DEGs) implicated in stress signaling, detect salt-responsive metabolic changes such as osmolytes, phenolic acids, and amino acid derivatives, and explore associations between transcriptional regulation and metabolic reprogramming under salt stress. Together, these findings provide a multi-omics overview of maize shoot responses to salinity and highlight candidate genes and metabolites for future functional validation and molecular breeding of salt-tolerant varieties.

## 2. Results

### 2.1. Salt-Stress-Induced Changes in Maize Growth and Physiology

**Figure 1:**
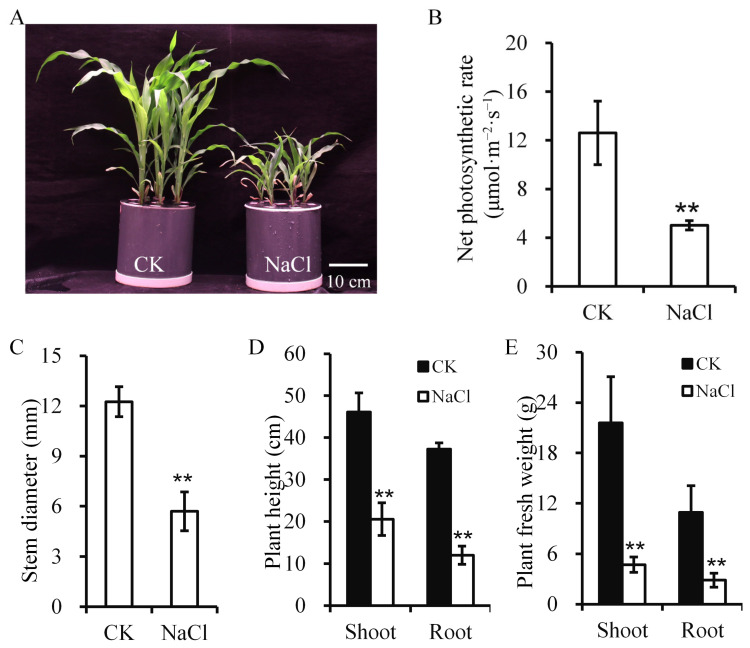
Maize phenotypic and physiological alterations under salt stress. (**A**) Plant phenotype. Scale bar = 10 cm. (**B**) Net photosynthetic rate. (**C**–**E**) Stem diameter (**C**), plant height (**D**), and fresh weight (**E**). Asterisks denote significance (** *p* < 0.01) vs. control. CK, control; NaCl, salt stress.

To evaluate how salinity affects maize development, plants were treated with NaCl and key growth and physiological parameters were analyzed. Plant height, net photosynthetic rate, and biomass were measured. Under the saline condition, the leaf edges showed yellowing or browning and older leaves appeared dry or necrotic (Figure 1A). The net photosynthetic rate declined sharply under salt stress (Figure 1B). Twelve days of salt stress markedly suppressed maize growth, with reductions in plant height, stem diameter, and fresh weight (Figure 1C–E). Plant height began to decline after six days of treatment, and plant fresh weight was reduced by 21.84% after 12 days of treatment (Figure S1 and Figure 1E).

### 2.2. Mineral Elements Content Responses to Salt Stress

**Figure 2:**
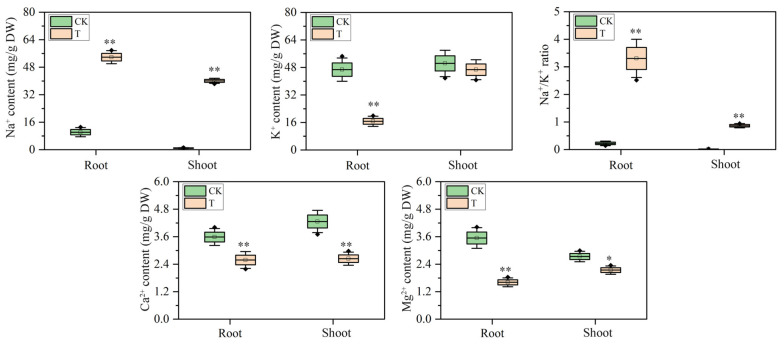
Ion content in maize under salt stress. Asterisks denote significance (* *p* < 0.05, ** *p* < 0.01) vs. control. CK, control; NaCl, salt stress.

To clarify the ion-specific responses to salinity, the ion concentrations were quantified in both shoots and roots. The results revealed significant changes in ion concentrations under salt stress (Figure 2). Na^+^ content increased substantially in both roots and shoots, with 4.28-fold and 42.96-fold elevations, respectively. This accumulation reflects maize’s response to salinity and may cause osmotic stress and ionic imbalance. K^+^ concentration declined by 64.28% in shoots, whereas root K^+^ levels remained relatively stable. Therefore, the Na^+^/K^+^ ratio rose in both tissues. The ratio increased from 0.02 to 0.86 in shoots and from 0.22 to 3.31 in roots, indicative of severe ionic imbalance. Both Ca^2+^ and Mg^2+^ concentrations were also reduced. Ca^2+^ declined by 38.07% in shoots and 27.93% in roots. Mg^2+^ decreased by 21.65% in shoots and 54.56% in roots.

### 2.3. Antioxidative Enzyme Responses to Salt Stress

**Figure 3:**
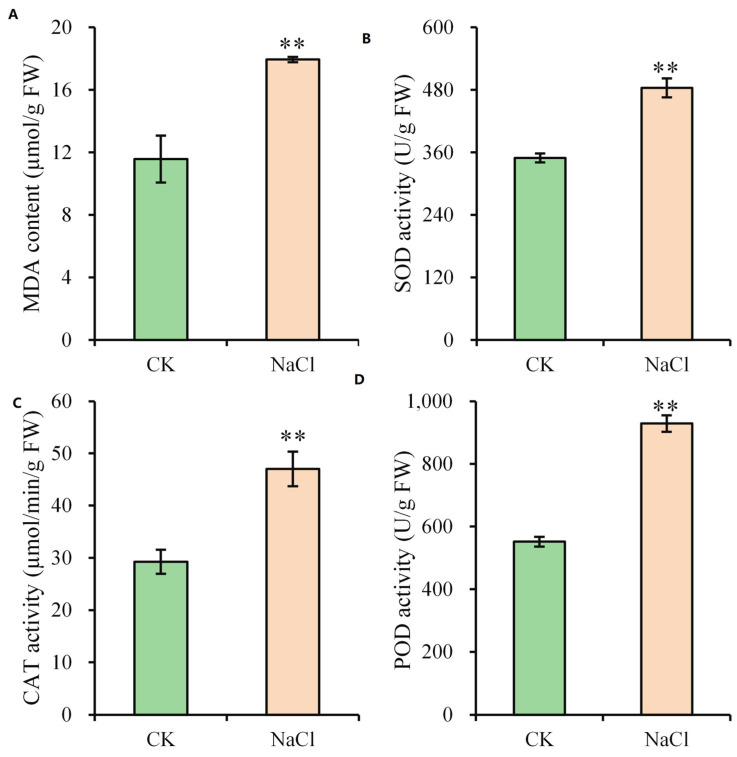
Biochemical responses of maize to salt stress. (**A**) MDA content. (**B**–**D**) Activity of antioxidant enzymes SOD (**B**), CAT (**C**), and POD (**D**). Asterisks denote significance (** *p* < 0.01) vs. control. CK, control; NaCl, salt stress.

Given the growth inhibition, leaf necrosis, and altered ion content, malondialdehyde (MDA) content and key antioxidant enzyme activities were assessed. Salt stress increased MDA content by 55.06% (Figure 3), which indicates oxidative damage to cellular membranes and membrane lipid peroxidation. The activities of peroxidase (POD), catalase (CAT), and superoxide dismutase (SOD) were quantified to examine antioxidant defense response. Their activities rose by 68.48%, 60.70%, and 38.41%, respectively, in leaves compared to the control (Figure 3).

### 2.4. Transcriptomic Profiling in Leaves Under Salt Stress

**Figure 4:**
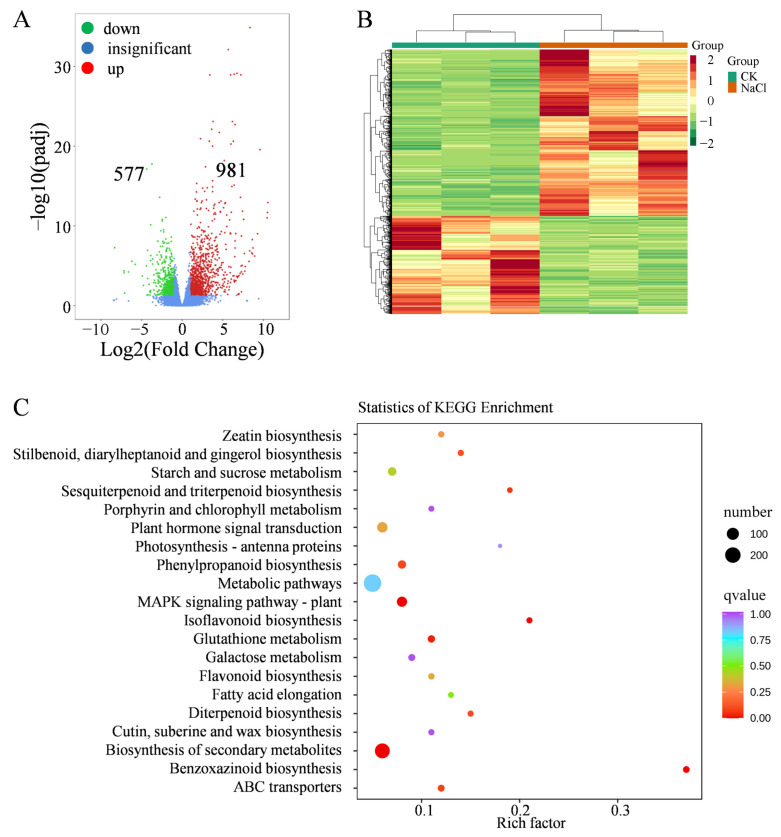
Transcriptomic analysis of maize under salt stress. (**A**) Statistics of differentially expressed genes (DEGs). (**B**) Cluster analysis of DEGs. (**C**) KEGG pathway enrichment analysis of DEGs.

To investigate maize shoot salt-stress-induced transcriptional responses, RNA sequencing was performed. All samples produced high-quality transcriptome data, with more than 94% of raw reads retained as clean reads, Q30 scores above 94.4%, GC contents between 55.9% and 57.3%, and a low error rate of 0.02% (Table S1). RNA-seq identified 1558 DEGs, including 981 upregulated and 577 downregulated genes (Figure 4A, Table S2). Clustering patterns clearly separated salt-treated samples from controls (Figure 4B). In molecular function categories, DEGs were enriched in heme binding, oxidoreductase activity, iron ion binding, and glucosyltransferase activity (Table S2). Biological processes included secondary metabolic processes, responses to antibiotics or toxic substances, cation transport, and metal ion transport (Table S3). KEGG analysis highlighted enrichment in multiple pathways, including secondary metabolite biosynthesis, MAPK signaling, ABC transporters, iso-flavonoid biosynthesis, glutathione metabolism, and phenylpropanoid biosynthesis (Figure 4C). Key genes in the MAPK signaling pathway (e.g., *PYR/PYL*, *PP2Cs*, *MAPK*, and *ANP1*) were differentially expressed (Figure S2A), which may influence ROS production, stomatal behavior, and stress adaptation. Genes involved in plant hormone (auxin, cytokinin, gibberellin, abscisic acid, ethylene, and brassinosteroid) signal transduction pathway were also regulated (Figure S2B). In addition, 71 transcription factors (TFs) showed altered expression (Figure S3, Table S4). These TFs included MYB, bHLH, NAC, AP2, WRKY, and bZIP, suggesting their regulatory roles in metabolic and stress-responsive pathways.

### 2.5. Metabolomic Analysis in Leaves Under Salt Stress

**Figure 5:**
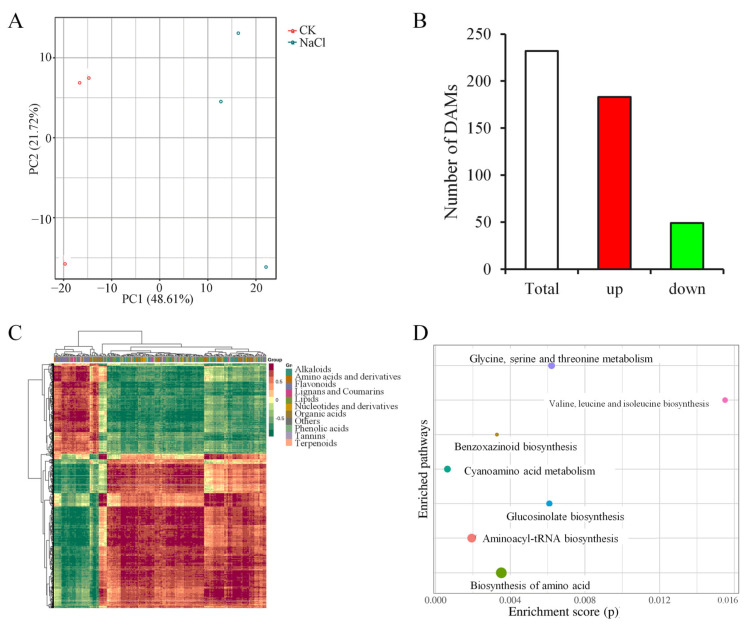
Metabolomic profiling of maize under salt stress. (**A**) PCA of metabolites under salt stress treatment. (**B**) Numbers of upregulated and downregulated differentially accumulated metabolites (DAMs). (**C**) Clustering heatmap of DAMs. (**D**) KEGG pathway enrichment analysis of DAMs.

Metabolomic profiling was conducted to complement the transcriptomic results. The analysis identified differentially accumulated metabolites (DAMs) between the control and salt-stressed maize shoots. Principal component analysis (PCA) separated the metabolic profiles between control and salt-stressed maize shoots along the first principal component (PC1) (Figure 5A). In total, 232 DAMs were identified in the NaCl-treated group, with 183 showing increased accumulation and 49 showing decreased accumulation (Figure 5B, Table S5). These metabolites were classified into alkaloids (38), flavonoids (35), phenolic acids (34), amino acids and derivatives (30), organic acids (24), lipids (23), nucleotides and derivatives (14), lignans and coumarins (13), terpenoids (5), tannins (2), and several other metabolites (Figure 5C). These classifications help clarify how salt stress alters metabolic networks in maize. Enrichment analysis showed significant activity in pathways such as aminoacyl-tRNA, benzoxazinoid, glucosinolate, and amino acid biosynthesis, as well as glycine/serine/threonine and cyanoamino acid metabolism (Figure 5D, Table S6).

**Figure 6:**
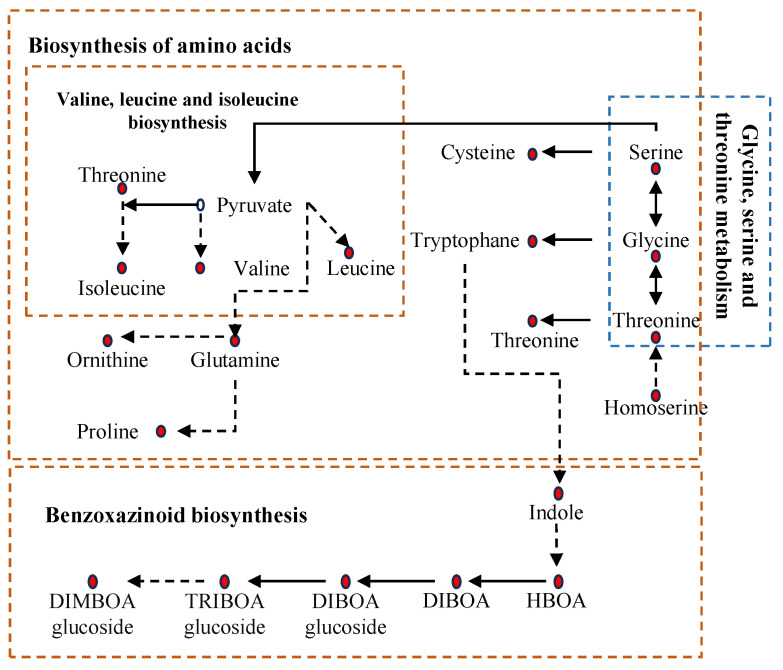
Heatmap representation of metabolites associated with amino acid biosynthesis, benzoxazinoid, and glycine/serine/threonine metabolism pathways. Red circles indicate accumulated metabolites.

Integrated transcriptomic and metabolomic analysis revealed coordinated molecular and biochemical changes. Transcriptional changes in amino acid biosynthesis pathways correlated with elevated levels of the corresponding metabolites (Table S3, Figure 6). Similarly, gene expression associated with glycine/serine/threonine metabolism also matched the increased accumulation of these amino acids. The transcriptional induction of benzoxazinoid biosynthetic genes corresponded with a marked rise in benzoxazinoid metabolites (Figure 4C and Figure 6).

**Figure 7:**
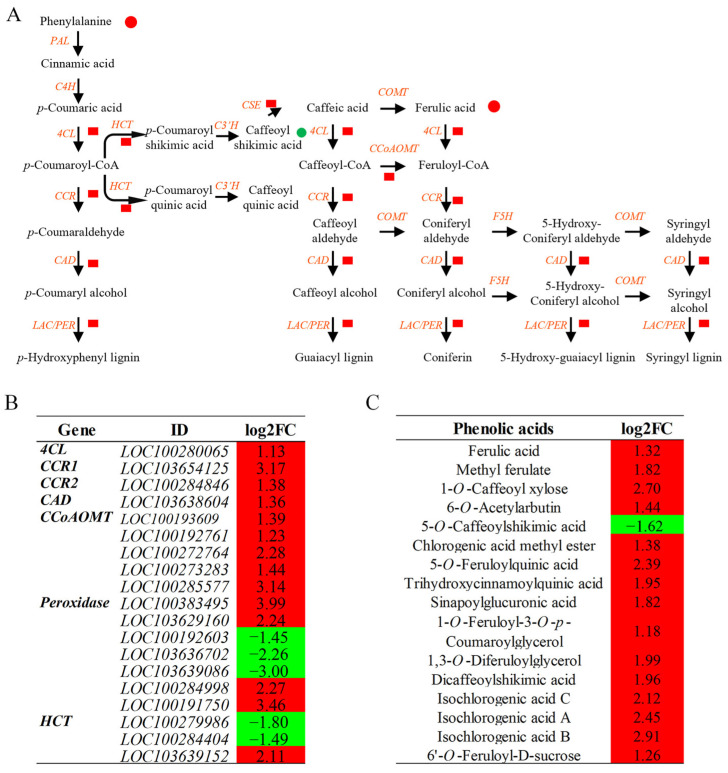
Alterations in phenolic acid metabolism under salt stress. (**A**) Schematic representation of metabolic changes: red and green circles indicate increased and decreased metabolites, respectively; red squares indicate upregulated genes. (**B**) DEGs related to phenolic acid metabolism. (**C**) DAMs related to phenolic acid metabolism.

Phenolic acid metabolism also showed pronounced changes (Figure 7). Ferulic acid accumulation was observed, and key genes in this pathway were upregulated, including *4CL* (4-coumarate–CoA ligase), *CAD* (cinnamyl alcohol dehydrogenase), *CCoAMT*, *CCR* (cinnamoyl-CoA reductase), and *CSE* (caffeoyl-CoA esterase). Several other phenolic acids also increased in content. Transcript abundances for representative genes in phenolic acid metabolism were validated by qRT-PCR, showing strong agreement with RNA-seq data (Figure S4). High-stringency correlation analysis was conducted to assess associations between DEGs and DAMs (|r| > 0.8, *p* < 0.05). Although these associations highlight coordinated responses, the temporal order of events and upstream regulatory mechanisms require time-series data to be resolved. These results present consistent multi-omics evidence but form a foundation rather than a complete regulatory model.

## 3. Discussion

Salt stress is a primary stress limiting maize growth, development, and yield. Identifying key responsive components and potential regulatory associations is essential for developing salt-tolerant maize cultivars. This study integrates phenotypic, physiological, and multi-omics analyses to characterize systemic maize shoots responses to salt stress and provides a unified view of stress adaptation.

### 3.1. Integrated Phenotypic–Physiological–Omics Responses to Salt Stress

Maize seedlings are affected by salt stress, especially during early growth [6]. Here, salt stress significantly inhibited maize seedling growth, resulting in reduced plant height, thinner stems, and leaf chlorosis. These phenotypic changes coincided with a sharp decline in photosynthetic rate and Na^+^ accumulation in plant tissues. The Na^+^/K^+^ ratio also increased. Elevated Na^+^ levels and a high Na^+^/K^+^ ratio disrupt ionic homeostasis and reduce photosynthetic efficiency, which ultimately limits biomass accumulation and future yield potential [14,15,16].

Salt stress further altered essential nutrient balance. Ca^2+^ and Mg^2+^ contents decreased in both tissues. Young seedlings, which have limited osmotic adjustment capacity and less efficient ion compartmentation, are especially vulnerable to these imbalances [30]. Combined with oxidative stress, these disturbances increased lipid peroxidation, reflected by higher MDA levels [12]. These findings suggest that salt stress challenges photosynthetic integrity and redox balance, and that seedlings may initiate protective adjustments to reduce physiological damage [30].

A notable observation was the reduction of K^+^ content in roots, whereas shoot K^+^ remained relatively stable. This pattern aligns with research showing that salt-tolerant lines maintain shoot K^+^ levels under stress [14]. K^+^ is essential for osmotic potential regulation, guard-cell function, enzyme activity, and directional growth responses [14,31]. Roots may act as a reservoir by mobilizing vacuolar K^+^ to preserve cytosolic K^+^ stability and sustain xylem loading. This spatial regulation may help protect shoot function and represent an adaptive strategy under salt stress.

### 3.2. Transcriptional Reprogramming to Salt Stress

Maize exhibited significant transcriptional reprogramming under salt stress, with 1558 DEGs identified in shoots. These DEGs were enriched in MAPK signaling pathways and secondary metabolism. MAPK cascades mediate key phosphorylation events that activate downstream TFs and stress-responsive genes [20]. Hormone signal transduction involving ABA, IAA, and ethylene also plays central roles in salt responses. Our results showed concurrent induction of core ABA signaling components, including ZmPYL and ZmPP2C, which may contribute to stress memory. Moreover, activation of MAPK cascades mediates key hormone signal transduction events, such as ABA signaling, during plant adaptation to abiotic stresses [32]. Activation of signaling pathways helps plants perceive and respond to salt stress, thereby initiating a series of defense mechanisms. This supports the idea that stress-resilient genotypes activate targeted regulatory networks rather than widespread transcriptional activity. Maize uses complex pathways to maintain or adapt to salt stress [15]. Like their responses to salt stress in shoots, where signal transduction and adaptive pathways also contribute to stress responses [15]. DEGs in roots are also enriched in MAPK signaling, and carbon metabolism, which have been described as adaptive pathways under salt stress [15,26,33]. Our analysis also showed that salinity regulated key nitrogen-related genes (nitrate reductase, glutamate synthase, nitrite transporter, and glutamine synthetase; Table S2). Since MAPKs such as RAF14/79 influence nitrogen signaling, this enrichment suggests crosstalk between stress and nutrient pathways, and reflects resource allocation toward osmolyte production and stress defense [34].

Several TF families were strongly regulated, including bHLH, NAC, AP2, MYB, and bZIP. *MYB* genes accounted for 15% of the regulated TFs, and *LOC100281900* and *LOC100280998* were notably repressed in shoots under salt stress (Table S4). We also identified *ZmPIF* genes (*LOC100382992*, *LOC100384229*, and *LOC100192921*) as strongly regulated, and these genes correlate with the gibberellin signaling pathway. These TF families are known to regulate antioxidant defense, osmotic adjustment, and hormone signaling. MYB, WRKY, and bHLH TFs contribute to secondary metabolism and antioxidant capacity, helping maintain redox and osmotic balance [35,36,37]. *ZmMYBR24* has been linked to flavonoid biosynthesis, regulating plant defense against salt stress [36]. *ZmbHLH32* is induced after salt treatment and enhances maize salt tolerance by activating *ZmIAA9* expression [35]. The regulation of both bHLH/WRKY TFs and nitrogen-related genes suggests that these TF families may mediate crosstalk between stress and nitrogen pathways, strengthening adaptive resource reallocation [38]. Supporting this, *Arabidopsis WRKY1* directly activates nitrogen assimilation and transport genes, integrating nitrogen remobilization with flowering and leaf senescence [39]. The co-enrichment of MAPK signaling and secondary metabolism indicates that TF-mediated signaling cascades may act as conserved regulators linking stress perception to metabolic adjustment [15].

### 3.3. Metabolic Reprogramming and Its Coordination with Transcriptional Changes

The combined metabolomic and transcriptomic analyses revealed broad metabolic reprogramming in maize shoots under salt stress. Affected metabolites such as amino acids and derivatives, phenolic acids, and lipids contribute to osmotic balance, ROS detoxification, and membrane stability [40]. Regulation of these pathways supports maize adaptation to salt stress [41,42,43,44,45]. Accumulated amino acids including proline and glycine betaine can contribute to osmotic balance and ROS detoxification [41]. Phenolic compounds, including ferulic acid and caffeoyl derivatives, were also increased and may strengthen antioxidant capacity [43]. Organic acids help regulate intracellular pH and ion balance [45], whereas adjustments in lipid metabolism likely support membrane structure under stress conditions [44]. These metabolic changes aligned with transcriptional shifts in the corresponding pathways. However, the direct regulatory relationships, including the control of metabolic genes by specific TFs, remain insufficiently characterized. Functional validation and network modeling will be important to clarify these connections.

This study presents an integrated view of maize seedling responses to salt stress. The results highlight the importance of ionic balance, redox regulation, and metabolic adjustment in stress adaptation. Although coordinated transcriptional and metabolic changes were identified, the single-genotype design limits the resolution of dynamic regulatory hierarchies. The underlying regulatory mechanisms still require investigation, especially the roles of individual TFs in regulating metabolic pathways. Comparative studies across maize genotypes may help identify conserved and genotype-dependent mechanisms and support future breeding salt-tolerant cultivars.

## 4. Materials and Methods

### 4.1. Plant Materials and Salt Stress Treatment

Maize inbred line Z58 was used. It is the female parent of Zhengdan958, a widely cultivated hybrid in northern China. Surface-sterilized seeds (10% NaClO, 15 min) were washed with water and they were germinated in coarse quartz sand. Two-leaf stage seedlings were transferred and grown in a chamber (25 °C, 60% RH, 16:8 h light/dark cycle). After three days of growth, seedlings were treated with 150 mM NaCl, whereas control plants received NaCl-omitted modified Hoagland solution (CK) [46]. The solution was replaced every three days throughout the 12-day salt-treatment period. Upon completion, shoot tissues were collected for integrated transcriptomic and metabolomic profiling. Three biological replicates were used.

### 4.2. Measurement of Leaf Photosynthetic Rate

Following the 12-day treatment period, photosynthetic parameters were quantified with an LI-6800 portable photosynthesis system (LI-COR Inc., Lincoln, NE, USA). A constant photosynthetically active radiation (800 μmol·m^−2^·s^−1^) was applied during all measurements. The uppermost fully expanded leaves were evaluated. Three independent biological replicates were analyzed.

### 4.3. Measurement of Ion Concentration

Ion concentration was examined by inductively coupled plasma mass spectrometry (ICP-MS). Dried shoot samples (0.08 g) were digested in polytetrafluoroethylene (PTFE) vessels with 5 mL nitric acid and incubated overnight. The vessels were then heated at 80 °C (2 h), 120 °C (2 h), and 160 °C (4 h) in a constant-temperature oven. After cooling, the digestate was evaporated to near dryness, dissolved in 1% nitric acid, and diluted to 25 mL. Elemental quantification was performed by ICP-MS using *m*/*z* detection and internal standardization. A reagent blank was processed for quality control. Three independent replicates were analyzed.

### 4.4. Quantification of Antioxidant Enzyme Activities and MDA Content

Cold extraction buffer (50 mM phosphate buffer (pH 7.8), 0.2 mM EDTA, 2% polyvinylpyrrolidone, and 2 mM L-ascorbic acid) were used to receive homogenized samples. From this, centrifugation (12,000× *g*, 10 min, 4 °C) produced a supernatant wherein all assays were performed. CAT activity was measured by the ammonium molybdate method, SOD activity by the nitroblue tetrazolium (NBT) method, POD activity by the guaiacol method, and MDA content was assessed through the thiobarbituric acid (TBA) reaction. All procedures followed manufacturer specifications (Cominbio Co., Suzhou, China), with three independent replicates.

### 4.5. RNA Isolation, Transcriptome Analysis and qRT-PCR Analysis

Total RNA isolation employed Trizol reagent (Invitrogen, Carlsbad, CA, USA), with its quality verified with a Nanodrop 2000 spectrophotometer (Thermo, Waltham, MA, USA). RNA sequencing libraries were prepared, each treatment represented by three independent replicates. Reads were sequenced on an Illumina platform (HiSeq-PE150) and aligned to the B73 genome (Zm-B73-REFERENCE-NAM-5.0) with HISAT2 (v2.0.5), and expression levels were normalized as RPKM. Differential expression analysis was obtained with DESeq2 (v1.22.1), considering |log_2_FC| ≥ 1 and FDR < 0.05 as significant. Functional annotation was conducted with Blast2GO (v3.0.8; e-value ≤ 1 × 10^−5^), and GO enrichment was analyzed with Goseq [47,48]. KEGG pathway annotation was performed using KAAS and visualized with clusterProfiler [49,50].

Following RNA extraction, cDNA was obtained from 0.5 μg RNA with the Vazyme R312 RT Kit. qRT-PCR with the SYBR Mixture system (Q411, Vazyme, Nanjing, China) was conducted on a Bio-Rad CFX96 system, with *ZmUBQ* (GRMZM2G066191) as reference. Expression levels from three biological replicates were calculated using the 2^−ΔΔCT^ method. Table S7 lists the primers.

### 4.6. Metabolite Measurements

Metabolite profiling was performed by Wuhan MetWare Biotechnology Co., Ltd. (Wuhan, China) [51,52]. Lyophilized shoot samples were powdered and subjected to extraction in a 70% aqueous methanol at 4 °C. This extraction approach captures comprehensive coverage of polar and semi-polar metabolites but has lower efficiency for non-polar lipids. Extracts were obtained following centrifugation (12,000× *g*, 10 min) and filtration (0.22 μm). UPLC-MS/MS (SHIMADZU Nexera X2 coupled to an Applied Biosystems 4500 QTRAP, Thermo, Waltham, MA, USA) was used for profiling. DAMs were defined using VIP ≥ 1 and |log_2_FC| ≥ 1.

### 4.7. Correlation Analysis of Transcript and Metabolite Pairs

Correlation analysis between DEGs and DAMs was performed with fold-change values. Pairs showing strong Pearson correlations with |r| > 0.8 (*p* < 0.05) were considered significantly associated.

### 4.8. Statistical Analysis

Values are presented as the mean ± SD. Inter-group differences (control vs. NaCl) were evaluated by Student’s *t*-test, adopting significance changes of *p* < 0.05 and *p* < 0.01.

## 5. Conclusions

**Figure 8:**
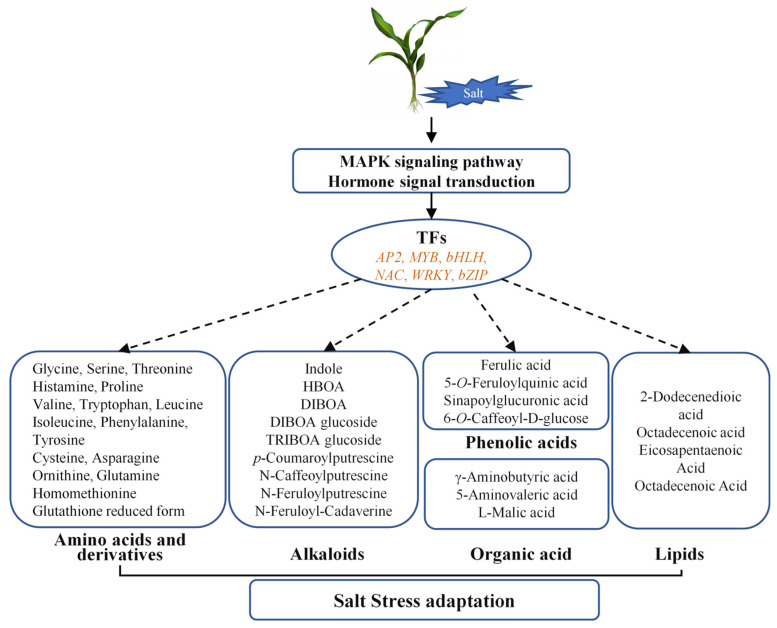
Hypothetical framework illustrating the shoot response mechanism of maize under salt stress.

This multi-omics study characterized shoot responses to salt stress in maize (Figure 8). Physiologically, salt stress significantly inhibited photosynthesis, reduced plant biomass, and severely disturbed ion homeostasis, evidenced by elevated Na^+^/K^+^ ratios and increased MDA content, while concurrently enhancing antioxidant enzyme activities. Transcriptomic profiling identified 1558 DEGs predominantly enriched under salt stress in maize shoots. Metabolomic analysis further revealed 232 DAMs. Coordinated transcriptional and metabolic adjustments were observed, including changes in genes associated with MAPK signaling, hormone transduction, and several TF families (AP2, bHLH, bZIP, MYB, NAC, WRKY), together with clear reprogramming of protective metabolic pathways. These correlative datasets provide a useful resource and highlight strong candidate genes and metabolites for future functional validation to clarify their roles within the regulatory network that supports salt tolerance in maize, thereby supporting the breeding of salt-tolerant varieties.

## Data Availability

All data generated in this study are available to the public and included in the article and its Supplementary Materials.

## Supplementary Materials

The following supporting information can be downloaded at https://www.mdpi.com/article/10.3390/plants14233554/s1. Table S1: Summary of maize shoot transcriptome sequencing and assembly quality; Table S2: Salt stress-responsive DEGs; Table S3: GO enrichment of DEGs; Table S4: Transcription factors among DEGs; Table S5: Salt stress-responsive DEGs; Table S6: KEGG enrichment of DAMs; Table S7: qRT-PCR primer sequences; Figure S1: Salt stress effect on shoot height; Figure S2: DEGs implicated in MAPK cascade (A) and hormone transduction (B); Figure S3: Differentially expressed transcription factors in salt-stressed maize shoots; Figure S4: qRT-PCR validation of expression in maize shoots.

## Abbreviations

The following abbreviations are used in this manuscript:

ABA	Abscisic Acid
CAT	Catalase
DAM	Differentially Accumulated Metabolite
DEG	Differentially Expressed Gene
GO	Gene Ontology
ICP-MS	Inductively Coupled Plasma Mass Spectrometry
KEGG	Kyoto Encyclopedia of Genes and Genomes
MAPK	Mitogen-Activated Protein Kinase
MDA	Malondialdehyde
NBT	Nitroblue Tetrazolium
PCA	Principal Component Analysis
POD	Peroxidase
ROS	Reactive Oxygen Species
SOD	Superoxide Dismutase
TF	Transcription Factor
TBA	Thiobarbituric Acid
UPLC-MS/MS	Ultra-Performance Liquid Chromatography–Tandem Mass Spectrometry

## References

[1] Landi S., Hausman J.F., Guerriero G., Esposito S. (2017). Poaceae vs. Abiotic stress: Focus on drought and salt stress, recent insights and perspectives. Front. Plant Sci..

[2] Munns R., Tester M. (2008). Mechanisms of salinity tolerance. Annu. Rev. Plant Biol..

[3] Zhang M., Liang X., Wang L., Cao Y., Song W., Shi J., Lai J., Jiang C. (2019). A HAK family Na^+^ transporter confers natural variation of salt tolerance in maize. Nat. Plants.

[4] Gill S.S., Tuteja N. (2010). Reactive oxygen species and antioxidant machinery in abiotic stress tolerance in crop plants. Plant Physiol. Biochem..

[5] Munns R., James R.A., Xu B., Athman A., Conn S.J., Jordans C., Byrt C.S., Hare R.A., Tyerman S.D., Tester M. (2012). Wheat grain yield on saline soils is improved by an ancestral Na^+^ transporter gene. Nat. Biotechnol..

[6] Farooq M., Hussain M., Wakeel A., Siddique K.H.M. (2015). Salt stress in maize: Effects, resistance mechanisms, and management. A review. Agron. Sustain. Dev..

[7] Zamani E., Bakhtari B., Razi H., Hildebrand D., Moghadam A., Alemzadeh A. (2024). Comparative morphological, physiological, and biochemical traits in sensitive and tolerant maize genotypes in response to salinity and Pb stress. Sci. Rep..

[8] Zhao K., Song J., Fan H., Zhou S., Zhao M. (2010). Growth response to ionic and osmotic stress of NaCl in salt-tolerant and salt-sensitive maize. J. Integr. Plant Biol..

[9] Hasanuzzaman M., Fujita M. (2022). Plant responses and tolerance to salt stress: Physiological and molecular interventions. Int. J. Mol. Sci..

[10] Farooq M., Zahra N., Ullah A., Nadeem F., Rehman A., Kapoor R., Al-Hinani M.S., Siddique K.H.M. (2024). Salt stress in wheat: Effects, tolerance mechanisms, and management. J. Soil. Sci. Plant Nutr..

[11] Wei G., Chen Y., Wang J., Feng L. (2024). Molecular cloning and characterization of farnesyl diphosphate synthase from *Rosa rugosa* Thunb associated with salinity stress. PeerJ.

[12] Kumar P., Choudhary M., Halder T., Prakash N.R., Singh V., Vineeth T.V., Sheoran S., Ravikiran K.T., Longmei N., Rakshit S. (2022). Salinity stress tolerance and omics approaches: Revisiting the progress and achievements in major cereal crops. Heredity.

[13] Shahid M.A., Sarkhosh A., Khan N., Balal R.M., Ali S., Rossi L., Gómez C., Mattson N., Nasim W., Garcia-Sanchez F. (2020). Insights into the physiological and biochemical impacts of salt stress on plant growth and development. Agronomy.

[14] Rizk M.S., Assaha D.V.M., Mekawy A.M.M., Shalaby N.E., Ramadan E.A., El-Tahan A.M., Ibrahim O.M., Metwelly H.I.F., Okla M.K., Maridueña-Zavala M.G. (2024). Comparative analysis of salinity tolerance mechanisms in two maize genotypes: Growth performance, ion regulation, and antioxidant responses. BMC Plant Biol..

[15] Maimaiti A., Gu W., Yu D., Guan Y., Qu J., Qin T., Wang H., Ren J., Zheng H., Wu P. (2025). Dynamic molecular regulation of salt stress responses in maize (*Zea mays* L.) seedlings. Front. Plant. Sci..

[16] Wang C., Wang Y., Cao X., Wu C., Wei X., Jiao P., Liu S., Ma Y., Guan S. (2025). Unraveling saline-alkali stress tolerance: Contrasting morpho-physiological, biochemical, and ionic responses in maize (*Zea mays* L.) genotypes. Plant Physiol. Biochem..

[17] Fang X., Mo J.J., Zhou H., Shen X., Xie Y., Xu J., Yang S. (2023). Comparative transcriptome analysis of gene responses of salt-tolerant and salt-sensitive rice cultivars to salt stress. Sci. Rep..

[18] Han Y., Wu C., Ji X., Yang M., Zhu H., Pei Z., Qu M., Qu L., Li Z., Yan S. (2025). Molecular mechanisms between salt-tolerant and salt-sensitive rice (*Oryza sativa* L.) varieties under salt stress. Curr. Issues Mol. Biol..

[19] Yu W., Wu W., Zhang N., Wang L., Wang Y., Wang B., Lan Q., Wang Y. (2022). Research advances on molecular mechanism of salt tolerance in *Suaeda*. Biology.

[20] Quan C., Huang S., Yu Q., Chen Z., Kashif M., Xu M., Wei F., Tang D. (2025). Physiological, biochemical, and transcriptomic analyses reveal potential candidate genes of *Platostoma palustre* in response to salt stress. BMC Plant Biol..

[21] Nakagami H., Pitzschke A., Hirt H. (2005). Emerging MAP kinase pathways in plant stress signalling. Trends Plant Sci..

[22] Kong X., Lv W., Zhang D., Jiang S., Zhang S., Li D. (2013). Genome-wide identification and analysis of expression profiles of maize mitogen-activated protein kinase kinase kinase. PLoS ONE.

[23] Kong X., Pan J., Zhang M., Xing X., Zhou Y., Liu Y., Li D., Li D. (2011). ZmMKK4, a novel group C mitogen-activated protein kinase kinase in maize (*Zea mays*), confers salt and cold tolerance in transgenic *Arabidopsis*. Plant Cell Environ..

[24] Cai G., Wang G., Wang L., Liu Y., Pan J., Li D. (2014). A maize mitogen-activated protein kinase kinase, ZmMKK1, positively regulated the salt and drought tolerance in transgenic. J. Plant Physiol..

[25] Zhang C., Chen B., Zhang P., Han Q., Zhao G., Zhao F. (2023). Comparative transcriptome analysis reveals the underlying response mechanism to salt stress in maize seedling roots. Metabolites.

[26] Reshi Z.A., Ahmad W., Lukatkin A.S., Bin Javed S. (2023). From nature to lab: A review of secondary metabolite biosynthetic pathways, environmental influences, and in vitro approaches. Metabolites.

[27] Wang C., Wei X., Wang Y., Wu C., Jiao P., Jiang Z., Liu S., Ma Y., Guan S. (2025). Metabolomics and transcriptomic analysis revealed the response mechanism of maize to saline-alkali stress. Plant Biotechnol. J..

[28] Liu Q., Kang J., Du L., Liu Z., Liang H., Wang K., He H., Zhang X., Wang Q., Hong Y. (2025). Single-cell multiome reveals root hair-specific responses to salt stress. New Phytol..

[29] Ren S., Bai T., Ma Y., Zhao Y., Ci J., Ren X., Zang Z., Ma C., Xiong R., Song X. (2025). Molecular mechanisms underlying salt tolerance in maize: A combined transcriptome and metabolome analysis. Plants.

[30] Ullah M.S., Mahmood A., Alawadi H.F.N., Seleiman M.F., Khan B.A., Javaid M.M., Wahid A., Abdullah F., Wasonga D.O. (2025). Silicon-mediated modulation of maize growth, metabolic responses, and antioxidant mechanisms under saline conditions. BMC Plant Biol..

[31] Chérel I., Gaillard I. (2019). The complex fine-tuning of K^+^ fluxes in plants in relation to osmotic and ionic abiotic stresses. Int. J. Mol. Sci..

[32] Danquah A., de Zelicourt A., Colcombet J., Hirt H. (2014). The role of ABA and MAPK signaling pathways in plant abiotic stress responses. Biotechnol. Adv..

[33] Sun Y., Zhao N., Sun H., Xu S., Lu Y., Xi H., Guo Z., Shi H. (2024). Transcriptome profiling reveals molecular responses to salt stress in common vetch (*Vicia sativa* L.). Plants.

[34] Gomez-Osuna A., Calatrava V., Galvan A., Fernandez E., Llamas A. (2020). Identification of the MAPK cascade and its relationship with nitrogen metabolism in the green alga *Chlamydomonas reinhardtii*. Int. J. Mol. Sci..

[35] Yan Z., Li K., Li Y., Wang W., Leng B., Yao G., Zhang F., Mu C., Liu X. (2023). The ZmbHLH32-ZmIAA9-ZmARF1 module regulates salt tolerance in maize. Int. J. Biol. Macromol..

[36] Bao L., Sun W., Wang J., Zhou Y., Wang J., Wang Q., Sun D.Q., Lin H., Fan J., Zhou Y. (2025). The transcription factor *ZmMYBR24* gene is involved in a variety of abiotic stresses in maize (*Zea mays* L.). Plants.

[37] Bo C., Cai R., Fang X., Wu H., Ma Z., Yuan H., Cheng B., Fan J., Ma Q. (2022). Transcription factor ZmWRKY20 interacts with ZmWRKY115 to repress expression of ZmbZIP111 for salt tolerance in maize. Plant J..

[38] Tian T., Wang J., Wang H., Cui J., Shi X., Song J., Li W., Zhong M., Qiu Y., Xu T. (2022). Nitrogen application alleviates salt stress by enhancing osmotic balance, ROS scavenging, and photosynthesis of rapeseed seedlings (*Brassica napus*). Plant Signal. Behav..

[39] Zhang W., Tang S., Li X., Chen Y., Li J., Wang Y., Bian R., Jin Y., Zhu X., Zhang K. (2024). *Arabidopsis WRKY1* promotes monocarpic senescence by integrative regulation of flowering, leaf senescence, and nitrogen remobilization. Mol. Plant..

[40] Vignesh P., Mahadevaiah C., Parimalan R., Valarmathi R., Dharshini S., Nisha S., Suresha G.S., Swathi S., Swamy H.K.M., Sreenivasa V. (2021). Comparative de novo transcriptome analysis identifies salinity stress responsive genes and metabolic pathways in sugarcane and its wild relative *Erianthus arundinaceus* [Retzius] Jeswiet. Sci. Rep..

[41] Hoque M.A., Banu M.N., Okuma E., Amako K., Nakamura Y., Shimoishi Y., Murata Y. (2007). Exogenous proline and glycinebetaine increase NaCl-induced ascorbate-glutathione cycle enzyme activities, and proline improves salt tolerance more than glycinebetaine in tobacco Bright Yellow-2 suspension-cultured cells. J. Plant Physiol..

[42] Guo R., Shi L., Yan C., Zhong X., Gu F., Liu Q., Xia X., Li H. (2017). Ionomic and metabolic responses to neutral salt or alkaline salt stresses in maize (*Zea mays* L.) seedlings. BMC Plant Biol..

[43] Sharma A., Shahzad B., Rehman A., Bhardwaj R., Landi M., Zheng B. (2019). Response of phenylpropanoid pathway and the role of polyphenols in plants under abiotic stress. Molecules.

[44] Pan L., Hu X., Liao L., Xu T., Sun Q., Tang M., Chen Z., Wang Z. (2023). Lipid metabolism and antioxidant system contribute to salinity tolerance in halophytic grass seashore paspalum in a tissue-specific manner. BMC Plant Biol..

[45] Ullah A., Ali I., Noor J., Zeng F.J., Bawazeer S., Eldin S.M., Asghar M.A., Javed H.H., Saleem K., Ullah S. (2023). Exogenous γ-aminobutyric acid (GABA) mitigated salinity-induced impairments in mungbean plants by regulating their nitrogen metabolism and antioxidant potential. Front. Plant Sci..

[46] Zhao M., Tai H., Sun S., Zhang F., Xu Y., Li W. (2012). Cloning and characterization of maize miRNAs involved in responses to nitrogen deficiency. PLoS ONE.

[47] Love M.I., Huber W., Anders S. (2014). Moderated estimation of fold change and dispersion for RNA-seq data with DESeq2. Genome Biol..

[48] Conesa A., Gotz S., Garcia-Gomez J.M., Terol J., Talon M., Robles M. (2005). Blast2GO: A universal tool for annotation, visualization and analysis in functional genomics research. Bioinformatics.

[49] Wei G., Chen Y., Wang M., Xi Y., Xu Y., Hussain H., Zhu K., Xu Y., Bai M., Wang J. (2024). Integrative application of metabolomics and transcriptomics provides new insights into carotenoid biosynthesis during *Rosa rugosa* hips ripening. Food Biosci..

[50] Mao X., Cai T., Olyarchuk J.G., Wei L. (2005). Automated genome annotation and pathway identification using the KEGG Orthology (KO) as a controlled vocabulary. Bioinformatics.

[51] Xiong W., Wang Y., Guo Y., Tang W., Zhao Y., Yang G., Pei Y., Chen J., Song X., Sun J. (2022). Transcriptional and metabolic responses of maize shoots to long-term potassium deficiency. Front. Plant Sci..

[52] Yan H., Nie Y., Cui K., Sun J. (2022). Integrative transcriptome and metabolome profiles reveal common and unique pathways involved in seed initial imbibition under artificial and natural salt stresses during germination of halophyte quinoa. Front. Plant Sci..

