# Supplementary material for: Correlative Transcriptome and Metabolome Analysis of the Maize Shoot Response to Salt Stress
Source: Plants (Basel). 2025 Nov 21;14(23):3554. doi: 10.3390/plants14233554 (PMC12694175; doi:10.3390/plants14233554)
Supplement: Supplementary file 1 [file plants-14-03554-s001.zip › Supplementary figures and tables.pdf]

Supplementary figures and tables

Table S1 Summary of maize shoot transcriptome sequencing and assembly quality.
Table S2 Salt stress-responsive DEGs.
Table S3 GO enrichment of DEGs.
Table S4 Transcription factors among DEGs.
Table S5 Salt stress-responsive DEGs.
Table S6 KEGG enrichment of DAMs.
Table S7 qRT-PCR primer sequences.
Figure S1 Salt stress effect on shoot height.
Figure S2 DEGs implicated in MAPK cascade (A) and hormone transduction (B).
Figure S3 Differentially expressed transcription factors in salt-stressed maize shoots.
Figure S4 qRT-PCR validation of expression in maize shoots.

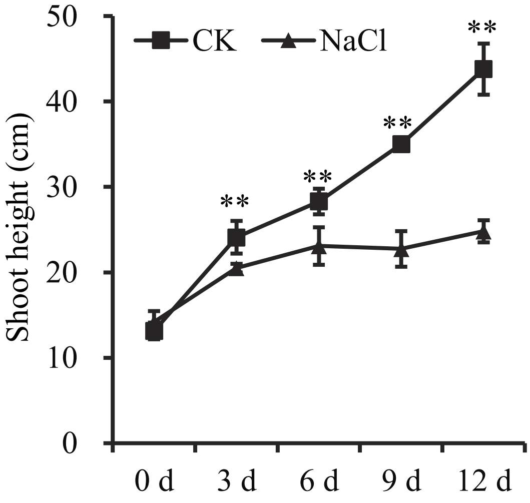

Figure S1 Salt stress effect on shoot height (***P* < 0.01).

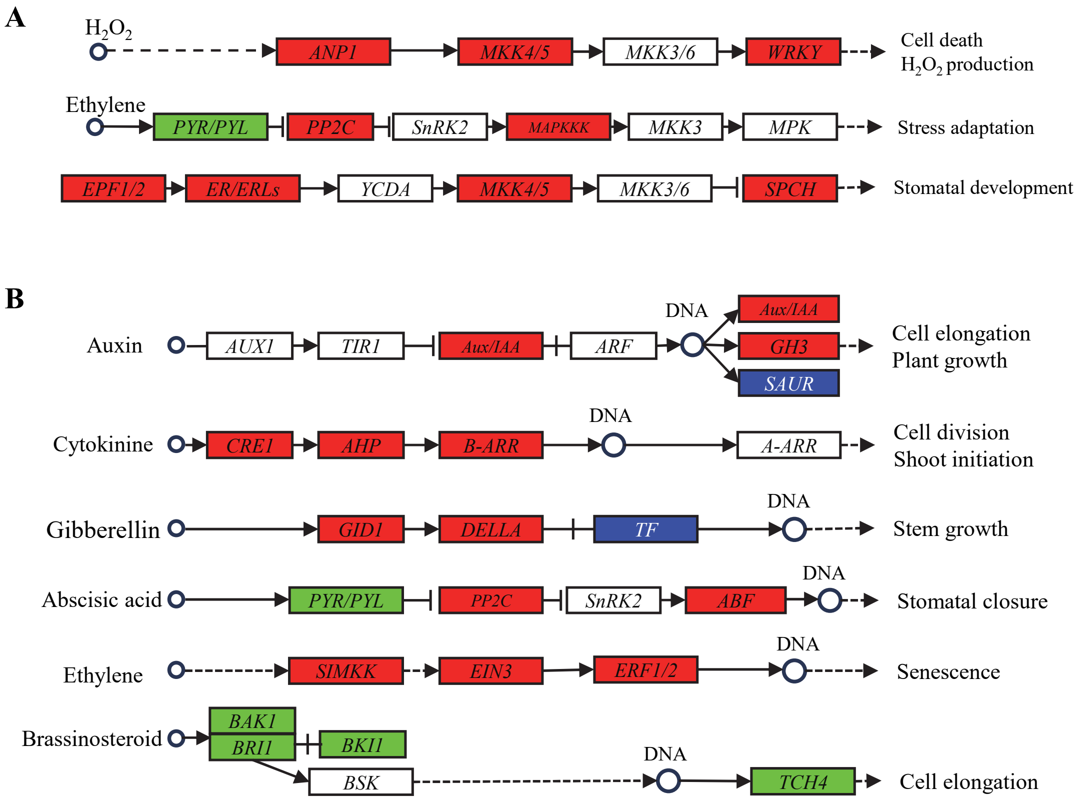

Figure S2 DEGs implicated in MAPK cascade (A) and hormone transduction (B).

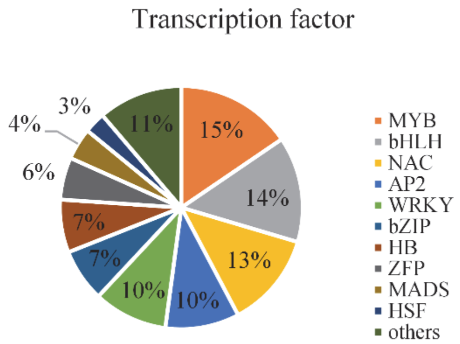

Figure S3 Differentially expressed transcription factors in salt-stressed maize shoots.

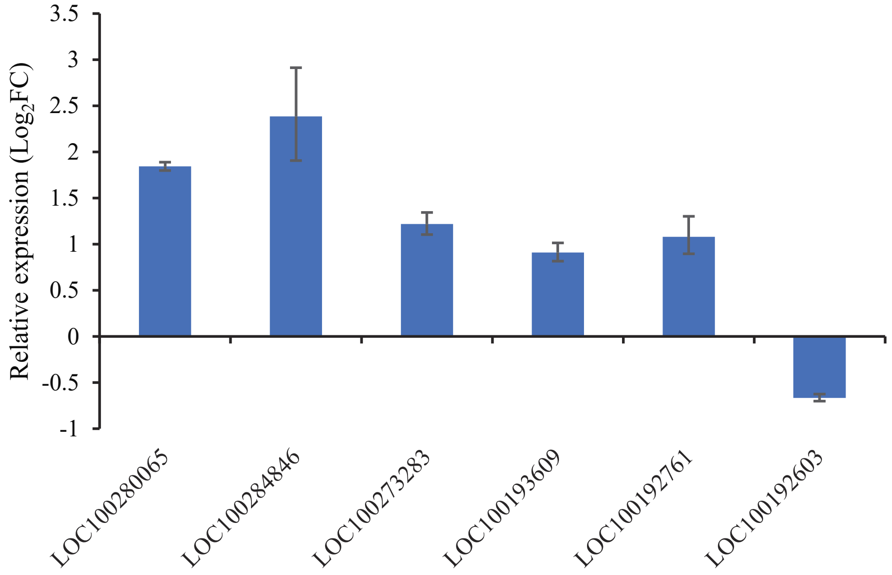

Figure S4 qRT-PCR validation of expression in maize shoots.
